# Vitamin B12 and Folate Status in Cognitively Healthy Older Adults and Associations with Cognitive Performance

**DOI:** 10.1007/s12603-020-1489-y

**Published:** 2020-10-13

**Authors:** L. Nalder, B. Zheng, G. Chiandet, L.T. Middleton, Celeste A. de Jager

**Affiliations:** 1 Ageing Epidemiology (AGE) Research Unit, School of Public Health, Imperial College London, London, UK; 2 Aged Persons Mental Health Program, NorthWest Mental Health, Melbourne, Victoria, Australia; 3 Faculty of Public Health and Policy, London School of Hygiene and Tropical Medicine, London, UK; 4 Public Health Directorate, Imperial College NHS Healthcare Trust, London, UK

**Keywords:** Vitamin B12 deficiency, folate, homocysteine, cognitive domains, RBANS, body mass index

## Abstract

**Objectives:**

To determine prevalence of vitamin B12 and folate deficiency and associations with cognitive performance in participants recruited for the Cognitive Health in Ageing Register: Investigational, Observational, and Trial Studies in Dementia Research: Prospective Readiness cOhort Study (CHARIOTPRO) SubStudy (CPRO-SS).

**Design:**

Cross-sectional analysis of data collected in the screening phase for the CPRO-SS.

**Setting:**

Participants were recruited from the Chariot Register at Imperial College London comprising approximately 39,000 community dwelling volunteers.

**Participants:**

Community dwelling individuals aged 60–85 years with B vitamin biomarker measures available were included (n=1946). After medical history and other exclusions, 1347 cognitively healthy participants were included for analysis of cognitive data.

**Measurements:**

Cognitive status was assessed with the Repeatable Battery for Neuropsychological Status (RBANS). Assays included vitamin B12 and folate, followed by serum methylmalonic acid and homocysteine levels for those with low vitamin B12. Gender-specific linear regression analysis was performed for associations between cognition and biomarkers. Non-gender specific regression for groups graded by B vitamin deficiency severity were also performed.

**Results:**

Vitamin B12 deficiency (<148pmol/L) was found in 17.2% of individuals and folate deficiency (<10nmol/L) in 1% of our participants. Low vitamin B12 was associated with poorer memory (p<0.03) in men. A high BMI predicted poorer attention and visuospatial indices (p<0.05). A regression analysis by B12 level revealed associations with poorer attention (β −6.46; p=0.004) for the deficient group and with immediate memory (β −2.99; p=0.019) for those categorised as severely deficient.

**Conclusion:**

Older men and women are prone to vitamin B12 deficiency with associated subtle and different domain-specific disruptive effects in measures of memory and attention. Elevated homocysteine and methylmalonic acid contributed to poorer cognitive performance. Novel groups at particular risk of cognitive deficit were identified for future interventional studies in this field.

## Background

The worldwide prevalence of B12 deficiency is estimated to be around 6%. However, prevalence is higher in adults over 60 years of age, ranging from 10% to 19% across various countries as reviewed by Smith et al (1) and others (2, 3, 4). The deficiency of folate is reported to be significantly rarer since the institution of fortification of grains in many countries world wide (5). The most recognised clinical manifestation of vitamin B12 and folate deficiency is that of megaloblastic anaemia (2). Given the emerging exponential increase of the ageing population and the significant global burden of dementia, its effect on cognitive performance globally and on individual cognitive domains require further elucidation, especially in cognitively healthy older adults who may be at risk of decline and dementia (6).

Common causes for B12 and folate deficiency include either low dietary intake of these macronutrients or poor absorption from the small intestine. Specific causative conditions include pernicious anaemia, terminal ileal disease or resection and adverse effects of common drugs such as metformin (7). Older adults and those at any age on vegetarian or vegan diets (8) without adequate supplementation are at risk of vitamin B12 deficiency. If not recognised and treated, these deficiencies may lead to a preventable cause of cognitive impairment (9).

There are two B 12-dependent enzymatic reactions important to maintaining health. These include methylmalonyl-CoA mutase conversion of methylmalonyl-CoA to succinyl-CoA and methylation of homocysteine (Hcy) to form methionine by methionine synthase. The latter reaction requires the presence of folate. Vitamin B12 deficiency will limit these cellular reactions resulting in increased concentrations of two key metabolic markers of B vitamin status, i.e. Hcy and methylmalonic acid (MMA), in the circulation (10). Elevated levels of Hcy and MMA and low levels of folic acid, all thought to be indicative of Vitamin B12 deficiency, have been reported to be associated with Alzheimer's disease (AD) and dementia (11). Vitamin B12 deficiency may accelerate cognitive decline (12) and can become an irreversible cause of dementia if left untreated for a prolonged period of time (1). Thus, older adults with B12 deficiency may have a reversible or preventable form of cognitive deterioration if identified and treated timeously.

The World Health Organisation traditionally defines the cutoff for serum B12 deficiency as below 148 pmol/L (SI), with the normal reference range from 148–600 pmol/L. However, the sharp cut-off between deficiency and normal vitamin B12 status may not be useful in terms of symptoms and treatment decisions as there is a continuous inverse relationship between serum B12 levels and a number of negative health outcomes, thus B12 insufficiency may also contribute to these (13).

Cognitive domains reported to be affected by poor B vitamin status are inconsistent, with Morris et al (12) previously suggesting that low circulating vitamin B12 was mostly associated with accelerated global cognitive decline, while low folate was associated more with memory deficits (14). Meanwhile, supra-normal folate status and high folic acid intake have been linked to slow information processing and accelerated decline in memory and global cognition (14). Clinical trials of B vitamin supplementation for older adults with and without dementia have given conflicting results with a lack of robust findings to support recommendations for prevention of cognitive decline (15). This in part may be due to small study samples, use of insensitive cognitive tests and metaanalyses combining results of studies with cognitively impaired and cognitively normal participants together.

Here, we present our cross-sectional data of B vitamin status and domain-sensitive cognitive test results collected from a large cohort of older community dwelling cognitively unimpaired volunteers. We aimed firstly to determine the prevalence of vitamin B12 and folate deficiency in our sample and, secondly, to evaluate associations of B12 and folate deficiency with global and domain-specific cognitive performance to indicate whether timely treatment may be warranted as a preventative measure against cognitive decline.

## Methods

### Study Population

Participants were recruited for inclusion in this cross sectional study from the biomarker-enriched Cognitive Health in Ageing Register: Investigational, Observational, and Trial Studies in Dementia Research: Prospective Readiness cOhort Study (CHARIOTPRO) SubStudy (CPRO-SS) (16) from the Chariot Register at Imperial College London (ICL). The Register was initiated in 2012 as a collaboration between the ICL School of Public Health and Primary Care practices in Greater London and now comprises >39,000 older community dwelling volunteers. A total of 2121 persons (n=2121) were screened and those aged 60 to 85 were included providing that they were willing and able to provide informed consent.. Participants were also required to be fluent in English and have adequate hearing and visual acuity to complete the required assessments. Exclusion criteria were: concurrent participation in an interventional trial; dementia or mild cognitive impairment (MCI) diagnosis; past or current use of memantine or Cholinesterase inhibitors; diagnosis of other neurologic disease or condition known to cause or be associated with dementia, such as Parkinson's disease; history of traumatic brain injury: history of stroke/diagnosis of transient ischemic attack (TIA), history of seizures: current diagnosis of significant psychiatric illness, as per DSM-IV, and currently in an acute phase/episode, or a current diagnosis or history of schizophrenia or bipolar disorder; history of hydrocephalus; uncontrolled hypo- or hyperthyroidism; chronic use of medications known to impair cognition; clinically significant infection within 30 days at study entry; self-reported HIV infection; history of alcohol or drug dependence or abuse as defined by (DSM IV-TR) criteria within the last 3 years. Cognitive screening was not applied for inclusion in this cross-sectional study.

A total of 1946 participants were selected based on availability of valid values in serum vitamin B12 and folate at screening for the prevalence analysis in this report. However, after medical exclusion criteria were applied some participants did not progress to have cognitive testing. Those who did progress to the next screening stage, with RBANS and no other missing data (n=1347), were included in the further analyses. Although none had a diagnosis of MCI or any dementia, some may have had subjective memory complaints or scored below the norms for age on the RBANS.

This study has received National Research Ethics Services approval and internal Imperial College London Research Ethics, Joint Research Compliance Office approval. All participants were provided with detailed study information on study procedures, risks and benefits and gave their informed consent.

### Study Procedures

At the first visit participants underwent medical and neurologic examination by a physician, including detailed medical history, biometrics and phlebotomy. Baseline data was collected on participant demographics (age, sex, educational attainment, height and weight for calculation of body mass index (BMI, kg/m^2^) and blood pressure (mmHg) and pulse (bpm). Blood samples were taken for screening biochemistry laboratory tests including cholesterol (SI), creatinine (SI), glucose (SI), Vitamin B12 and folate. For those whose vitamin B12 level was below 133pmol/L (n=220), plasma Hcy and MMA assays were performed to confirm deficiency (17, 18). Cognitive status was assessed by trained assistant psychologists with the Clinical Dementia Rating Scale [19] and the Repeatable Battery for Assessment of Neuropsychological Status (RBANS) (20).

### Cognitive tests

The RBANS is a 20 minute composite battery with 12 subtests that measure 5 cognitive domain indices: Attention: Digit Span and Coding subtests, Language: Picture Naming and Semantic Fluency, Visuospatial Construction: Figure Copy and Line Orientation, Immediate Memory: List Learning and Story Memory, Delayed Memory: List Recall, List Recognition, Story Recall, and Figure Recall subtests. The sum of the 5 Index scores is converted to a Total Scale value; a norm-based t score based on a distribution with a mean of 100 and standard deviation of 15. The RBANS has 3 alternate versions, and its utility has been demonstrated by the association with daily functional capacity in MCI patients, and with the CDR in both MCI and AD (21).

### Laboratory assay methods

Fasting serum samples were prepared from clotted blood and shipped frozen at -20 degrees Celsius to Covance Inc. Central Laboratory Service (CLS), Geneva (Switzerland) for chemi-luminescence assays of vitamin B12 and folate. For those whose results were below the normal range, further assays were performed for serum MMA and total EDTA plasma Hcy. Reference ranges were provided by Covance Inc CLS, Geneva (Vitamin B12: 133–675 pmol/L; Folate: >/=10 nmol/L; MMA: 0.0–0.40 *µ*mol/L; Hcy: 3.70–13.99 *µ*mol/L).

### Statistical Analysis

Descriptive statistics for biomarkers and demographic factors were compared between males and females using t-test or Mann-Whitney U test. Percentages of participants with vitamin B12 and folate levels below normal were determined for deficiency.

For analysis on associations of B12 and folate with cognitive test performance, we excluded participants with missing values in RBANS scores, BMI, and education level, and further excluded outliers for B12 (>1000 pmol/L) and folate (>80nmol/L). After exclusion, 1347 participants remained in the following analyses. Cognitive test performance was compared between males and females using t-tests. Correlations between RBANS scores and demographic variables (age, sex, education), biometrics and blood biomarkers were performed with Spearman's rho to determine which potentially confounding factors to include in the regression analyses (data not shown; relevant variables were included in the multiple linear regressions below).

Gender-specific multiple linear regression models were produced including folate, B12, BMI, age, and educational attainment as independent factors. Dependent factors included RBANS index scores by cognitive domain (Immediate and Delayed memory; Attention; Language; Visuospatial Construction) or by individual cognitive tests for each domain. We additionally adjusted for systolic and diastolic blood pressure, creatinine, cholesterol and glucose as a sensitivity analysis. The same multiple linear regression models were produced for men and women combined and presented as supplementary material (Table S1).

In addition, we generated a categorical variable combining information of all B12 biomarkers (normal: B12≥148 pmol/L; deficient: B12<148 pmol/L and folate≥10 nmol/L and Hcy≤ 13.99 *µ*mol/L and MMA≤ 0.4 *µ*mol/L; severely deficient: folate<10 nmol/L or Hcy>13.99 *µ*mol/L or MMA>0.4 *µ*mol/L in those with B12<148 pmol/L) and tested its association with RBANS indices using multiple linear regression. No gender-specific analysis was performed given small sample sizes of deficient groups. For all statistical analyses conducted, a p-value of <0.05 was interpreted as a statistically significant result.

## Results

Study participants had a mean ±SD age of 71.7 ±5.5 years. Approximately 40% were overweight (BMI 25–29.9kg/m^2^), 18.5% were obese (BMI ≥30kg/m^2^) and 1.2% underweight (BMI <18.5kg/m^2^). Demographic characteristics are presented by sex in Table 1. Females had higher vitamin B12, folate, cholesterol levels and pulse rate than males (all (p<0.001), but males were marginally older and had slightly higher systolic and diastolic blood pressure and significantly higher BMI and creatinine levels than females. For the subsample with B12<133 pmol/L, no differences in MMA and homocysteine levels were detected between men and women. There were differences in performance in some RBANS domain index scores between men and women within the normal range, with the mean scores for immediate and delayed memory scores being slightly higher in women, whilst, men had a higher mean score on Visuospatial Construction (P<0.001).

**Table 1:** Characteristics of study population by sex

**Characteristic**	**Male (n= 964)**	**Female (n= 982)**	**P value**
Age (years)	72.1 ± 5.5	71.4 ± 5.6	0.006
Educational attainment, n (%)*			0.001
Not completed secondary school	74 (9.7)	112 (14.3)	
Secondary school	99 (13.0)	111 (14.1)	
Tertiary education	102 (13.4)	105 (13.4)	
Bachelor's degree or equivalent	287 (37.6)	299 (38.1)	
Master's degree, equivalent or higher	201 (26.3)	158 (20.1)	
Folate (nmol/L)*	22.8 (14.1)	26.7 (18.9)	<0.001
B12 (pmol/L)*	199.5 (103.0)	232.0 (141.0)	<0.001
Hcy (*µ*mol/L)* (n=220)	15.7 (7.1)	15.0 (6.3)	0.483
MMA (*µ*mol/L)* (n=220)	0.22 (0.15)	0.24 (0.19)	0.120
BMI (kg/m^2^)	27.0 ± 3.6	25.9 ± 4.8	<0.001
Cholesterol (SI)	4.90 ± 1.02	5.72 ± 1.04	<0.001
Creatinine (SI)	86.1 ± 19.2	67.7 ± 12.3	<0.001
Glucose (SI)*	5.4 (0.9)	5.3 (1.0)	0.182
Systolic BP (mmHg)	140 ± 17	138 ± 20	0.030
Diastolic BP (mmHg)	80 ± 10	78 ± 10	0.009
Pulse (bpm)	65 ± 11	68 ± 10	<0.001
RBANS index scores	(n= 667)	(n= 680)	
Immediate Memory	100 ± 13	101 ± 13	0.012
Visuospatial Construction	98 ± 14	94 ± 16	<0.001
Language	103 ± 13	102 ± 13	0.107
Attention	108 ± 16	107 ± 15	0.258
Delayed Memory	97 ± 13	101 ± 11	<0.001
Total Scale	101 ±13	101 ± 13	0.931


Data shown as mean ± SD or median (IQR, interquartile range) unless otherwise specified; * Mann-Whitney U test was used due to the skewed distribution of these variables; Biomarker values are equivalent to SI Units; RBANS: Repeatable Battery for Assessment of Neuropsychological Status

The median value of of vitamin B12 (n=1946) was within the normal range at 215 pmol/L (interquartile range=125 pmol/L), with the median serum folate level at 24.7 nmol/L (interquartile range=16.5 nmol/L). Furthermore, figure la shows that a high percentage of participants (17.2%) had below threshold (deficient) vitamin B12 levels (<148pmol/L) and 35.1% had above threshold levels in the insufficient range between 149–220 pmol/L (fig la); only 1% had folate levels below the threshold of 10nmol/L (fig 1b).

**Figure 1b:**
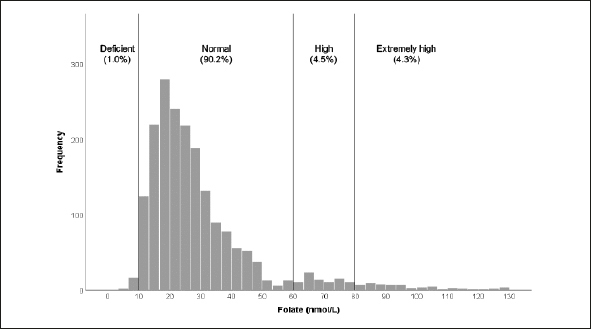
Folate distribution

**Figure 1a:**
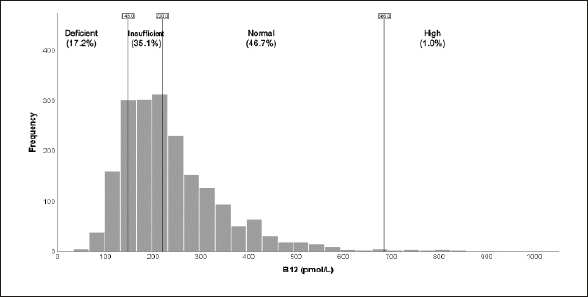
Vitamin B12 distribution

### Multivariate Linear Regression Models

*Comparison of vitamin B12, folate and BMI associations with RBANS cognitive domains and individual cognitive tests in males and females*

Linear regression models were, firstly, stratified by sex in view of the marginal differences described between males and females (fig 2a). No associations were found in women between vitamin B12 and any index scores or RBANS total scores; in men, there was a negative association between folate and Attention Index (−0.12 (95%CI: −0.22, −0.02), p=0.019); for every 1 nmol/L increase in folate, the Attention index decreases by 0.12 points.

**Figure 2a:**
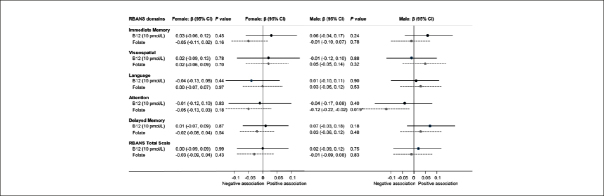
Multiple Linear Regression associations of vitamin B12 and folate with RBANS domain index scores Unstandardized regression coefficients (β-values) and 95% Confidence Intervals (CI) are presented. The results for vitamin B12 are scaled to 10pmol/L and folate as nmol/L. Thus, for every 10pmol/L increase in B12 the score will increase by the value of β. Significant associations are indicated with an * for those with P<0.05 and T for trend (P<0.10). Negative associations have β-values below 0 where higher cognitive scores are related to lower vitamin B12 or folate, and positive associations have β-values above 0, where higher (better) cognitive scores are related to higher B 12/folate. The results were adjusted for age, education and BMI.

Individual RBANS cognitive tests for each domain were entered into multiple linear regression models as dependent variables. The same model was used as for the index scores. (Figure 2b) Folate showed a negative association with the digit span test for females (p=0.009) and males (p=0.022), but in male participants, folate showed trends for positive associations with figure recall and picture naming (p<0.08); thus a high folate had negative effects on attention, but subtle positive effects on memory. Higher vitamin B12 levels predicted better immediate and delayed memory test scores (list learning, list recall and list recognition: p=0.023) for males. This effect was incremental; for every 100pmol/L increase in B12, list learning score increases by 0.4 points). These estimates did not change appreciably after potential confounding factors such as blood pressure, cholesterol, creatinine and glucose were included in the model (data not shown). Results for non gender-stratified regression showed subtle negative associations of folate with the attention index (p=0.015) and digit span scores (p=0.001) and trends (p<0.01) for associations of vitamin B12 levels with some memory scores (Supplementary Table 1).

**Figure 2b:**
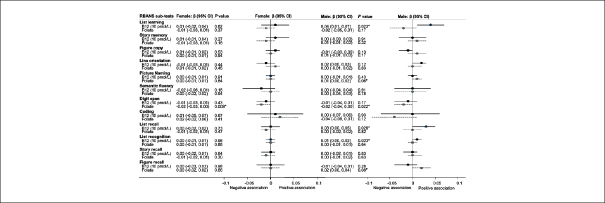
Multiple Linear Regression associations of vitamin B12 and folate with RBANS sub-test scores for females and males Unstandardized regression coefficients (β-values) and 95% Confidence Intervals (CI) are presented. The results for vitamin B12 are scaled to 10pmol/L and folate as nmol/L. Thus, for every 10pmol/L increase in B12 the score will increase by the value of β. Significant associations are indicated with an * for those wifh P<0.05 and T for trend (P<0.10). Negative associations have β-values below 0 where higher cognitive scores are related to lower vitamin B12 or folate, and positive associations have β-values above 0, where higher (better) cognitive scores are related to higher B12 or folate. The results were adjusted for age, education and BMI.

BMI was negatively associated with Attention Index, symbol digit coding and figure copy scores in men, and Visuospatial Index, figure copy, line orientation and digit span scores in women. (Table 2) Interaction terms for B12 and folate with BMI were also tested in the models. No B12×BMI interaction was detected (p>0.05), but there was a negative interaction for folate×BMI for digit span in women (p=0.002).

**Table 2:** Multiple linear regression associations between BMI and RBANS domain index and subtest scores for females and males

**RBANS Test scores**	**Female**		**Male**	
	**β (95% CI)**	**P**	**β (95% CI)**	**P**
Immediate Memory	−0.044 (−0.239, 0.151)	0.658	0.026 (−0.246, 0.298)	0.851
List learning	−0.008 (−0.073, 0.056)	0.796	0.029 (−0.056, 0.115)	0.499
Story memory	−0.024 (−0.074, 0.026)	0.344	−0.009 (−0.080, 0.061)	0.798
Visuospatial	−0.361 (−0.599, −0.124)	**0.003**	−0.269 (−0.556, 0.018)	0.066
Figure copy	−0.058 (−0.095, −0.021)	**0.002**	−0.050 (−0.099, −0.002)	**0.043**
Line orientation	−0.055 (−0.100, −0.009)	**0.019**	−0.012 (−0.060, 0.037)	0.641
Language	0.000 (−0.204, 0.203)	0.998	−0.135 (−0.411, 0.140)	0.335
Picture Naming	−0.008 (−0.023, 0.007)	0.315	−0.001 (−0.017, 0.015)	0.921
Semantic fluency	0.001 (−0.070, 0.072)	0.981	−0.046 (−0.145, 0.054)	0.368
Attention	−0.205 (−0.443, 0.032)	0.090	−0.323 (−0.644, −0.002)	**0.048**
Digit span	−0.043 (−0.082, −0.004)	**0.030**	−0.029 (−0.084, 0.026)	0.303
Coding	−0.045 (−0.172, 0.082)	0.487	−0.182 (−0.346, −0.018)	**0.030**
Delayed Memory	0.060 (−0.117, 0.237)	0.503	0.126 (−0.143, 0.395)	0.358
List recall	0.010 (−0.032, 0.053)	0.635	0.017 (−0.042, 0.075)	0.576
List recognition	0.014 (−0.003, 0.031)	0.097	0.011 (−0.018, 0.039)	0.460
Story recall	−0.004 (−0.036, 0.028)	0.822	−0.032 (−0.073, 0.010)	0.137
Figure recall	0.010 (−0.048, 0.068)	0.730	0.029 (−0.040, 0.098)	0.412
RBANS Total Score	−0.162 (−0.360, 0.036)	0.109	−0.162 (−0.428, 0.104)	0.233


Note: Adjusted for age, educational attainment, B12, and folate levels; CI= confidence interval; P<0.05 in bold type

### Associations of biomarker groups with cognition

After excluding B12 deficient subjects with missing values in Hcy or MMA, there remained 1103, 48, and 106 in the Normal, Deficient, and Severely deficient participant groups respectively available for analysis. When compared with the normal group, being in the Severely deficient group was associated with lower Immediate Memory indices (−2.995, CI −5.503, −0.488; p=0.019), and being in the Deficient group was associated with lower Attention indices (−6.462, CI −10.844, −2.080; p=0.004). (Table 3) There was a borderline significance for association of the Severely deficient group with lower total RBANS scores (−2.321 CI −4.812, 0.170; p=0.068).

**Table 3:** Associations between biomarker groups and RBANS domain index scores

**RBANS Sub Domains**	**Biomarker groups**				
	**Normal (Reference)**	**Deficient**		**Severely deficient**	
	**β**	**β (95% CI)**	**P**	**β (95% CI)**	**P**
Immediate Memory	0	−0.199 (−3.816, 3.418)	0.914	−2.995 (−5.503, −0.488)	**0.019**
Visuospatial	0	−0.925 (−5.089, 3.239)	0.663	−2.351 (−5.238, 0.535)	0.110
Language	0	1.743 (−2.010, 5.495)	0.362	−1.473 (−4.074, 1.128)	0.267
Attention	0	−6.462 (−10.844, −2.080)	**0.004**	−1.672 (−4.709, 1.366)	0.280
Delayed Memory	0	0.260 (−3.172, 3.692)	0.882	−0.078 (−2.457, 2.301)	0.949
RBANS Total Score	0	−1.495 (−5.089, 2.099)	0.415	−2.321 (−4.812, 0.170)	0.068


Note: Adjusted for age, sex, educational attainment, and BMI; CI= confidence interval; P<0.05 in bold type; Biomarker groups: Normal: B12≥148 pmol/L; Deficient: B12<148 pmol/L and folate≥10 nmol/L and Hcy≤ 13.99 *µ*mol/L and MMA≤ 0.4 *µ*mol/L; Severely deficient: folate<10 nmol/L or Hcy> 13.99 *µ*mol/L or MMA>0.4 *µ*mol/L in those with B12<148 pmol/L

## Discussion

In this prospective study of older community dwelling participants, we found the overall prevalence of vitamin B12 deficiency to be 17.2%, which although high, is in line with previously reported literature (1), We also found a high degree of B12 insufficiency/low normal status, as described by Smith & Refsum (13). Folate deficiency was negligible in our cohort even though folate fortification is still not mandatory in the UK. Women had better vitamin B12 status whilst having lower BMI and blood pressure measures than men. Low B12 levels were associated with high BMI and both factors independently predicted worse cognitive performance, whilst folate had both positive and negative effects on specific cognitive domains.

To our knowledge, this is the first study, in cognitively healthy older adults, to report on the specific cognitive domain differences associated with vitamin B12 deficiency between men and women. These differences are important to recognise in the clinical setting as awareness of them can help pave the way for early intervention for this treatable form of cognitive impairment. Previous reports of cognitive domains affected by poor vitamin B12 status in cognitively healthy older adults have been inconsistent and most have not drawn gender comparisons between the domains (14). One study found no effects of low B12 on global cognitive impairment (MMSE<24) for men or women (22), whilst another (23) found attention deficits for men & women combined. Whereas Morris (14) reported global cognitive decline related to low B12 we found domain-specific effects, with lower memory scores in men with low B12 and in our severely deficient group (low B12 plus low folate, elevated Hcy and/or MMA). In our deficient group, there was evidence of lower attention index scores. The conflicting findings may relate to the differences in sensitivity of cognitive outcome measures used, the prevalence of B12 deficiency or underlying confounders related to vascular health in the different study populations.

Our results suggest that older men and women with high BMI were potentially vulnerable in the cognitive domains of attention/executive function and visuospatial construction. The negative correlation between B12 and BMI and effects of both factors on attention and memory in B12 deficient older individuals have not been reported in previous literature. There are contrasting reports that high latelife BMI protects against AD and cognitive impairment (24) whilst high waist-to-hip ratio is associated with increased cognitive impairment (25). We add to this body of knowledge by presenting the largest cohort in which independent effects of both low B12 and high BMI reveal novel results identifying “within limits” domain-specific poorer cognitive performance.

Morris reported memory deficits with both low and supra-normal folate, as well as slower processing speed and global cognitive decline with supra-normal folate (14). We found a trend for better memory performance with higher folate, but worse attention with elevated folate in both men and women. These results are similar to the previously reported finding of a J-shaped dose-response relationship between folate exposure and risk of cognitive impairment for those with vitamin B12 deficiency (26). This may reflect the fact that high folate levels can mask B12 deficiency due to their shared metabolic reactions. This has clinical relevance with regard to the risk:benefits of food fortification with these vitamins (27).

Furthermore, we demonstrated a graded cognitive deterioration with increasing severity of vitamin B12 deficiency biomarkers (elevated Hcy/MMA) affecting more than one domain (memory and global performance), whilst those only B12 deficient had deficits restricted to the attention domain. These findings reflect previous literature reporting hyperhomocysteinemia as a known risk factor for cognitive impairment (9, 28) and cognitive decline in healthy older adults (29). This is of particular clinical relevance as previous intervention trials of B vitamin supplementation for cognitively normal older adults have failed to reveal consistent benefits of treatment (15). However, when targeting populations with elevated Hcy levels, benefits were seen in individuals with normal cognition as well as in those with mild cognitive impairment (30, 31, 32). Hcy-lowering and a higher methionione: Hcy ratio have also been associated with reduced rates of whole brain atrophy (32, 33).

Although relevant confounders were corrected for, there are limitations to our study worth consideration. Firstly, we performed multiple testings with the RBANS domain indices and subtest scores as outcome measures, which may have lead to an inflated Type-1 error rate. Since this is an exploratory study, our findings could be informative for future validation studies in cognitively healthy older adults. Secondly, as this was a cross-sectional study we can't make assumptions about B12 deficiency as the only cause of cognitive decline. Other factors, such as dietary deficiencies and alcohol consumption may effect cognitive function and also cause macronutrient deficiencies. Information on these factors was not collected in our study. Another limitation was the lack of biomarker data on Hcy and MMA for the full study cohort which limited a robust analysis of associations with cognition. Lastly, it could be argued that the cognitive deficits identified may be the cause rather than the result of macronutrient deficiency. The cognitive changes are, however, very specific and require a sensitive neuropsychological test battery to elicit. Therefore, they are unlikely to be severe enough to themselves cause a nutritional deficiency in this cohort of cognitively healthy participants.

We herein present novel data from a large sample of healthy older volunteers suggesting that vitamin B12 deficiency may have subtly different cognitive implications in men compared to women. Importantly, elevated BMI and low circulating vitamin B12 were independent risk factors for disruption in attention/executive, visuospatial skills and memory processes. Participants with high MMA and/or Hcy levels combined with low B12 had a higher chance of displaying poor memory and global cognitive performance. In addition, supranormal folate was associated with poorer attention in both sexes. These results are generalizable to cognitively healthy older adults in western countries. Future research in this field would benefit from further delineating any causative associations between BMI and B12 levels, as well as implementing therapeutic strategies by way of randomised controlled trials in those identified as at risk of cognitive impairment from B12 deficiency, to assess whether the subtle cognitive changes found in this population can be reversed.

### Key Points

• Older adults are prone to vitamin B12 deficiency, likely due to poor absorption or low dietary intake
• Specific cognitive domains associated with low vitamin B12 and high folate levels were found to be attention and immediate and delayed memory. These were affected in a graded fashion with regard to the severity of B12 deficiency.
• Men and women displayed differences in cognitive performances relating to vitamin B12 and folate status; men had poorer memory scores with lower B12.
• Severe B12 deficiency with additional abnormal biomarkers contributed to poorer immediate memory performance relative to normal B12.
• High body mass index (BMI) had negative effects on attention and visuospatial skills in men and women.

## Electronic Supplementary Material

TABLE S1. Non-gender stratified multiple linear regression for associations of RBANS domain index and subtest scores with vitamin B12 and folate
