# Supplementary material for: Vitamin B12 and Folate Status in Cognitively Healthy Older Adults and Associations with Cognitive Performance
Source: J Nutr Health Aging. 2020 Oct 13;25(3):287–94. doi: 10.1007/s12603-020-1489-y (PMC12280608; doi:10.1007/s12603-020-1489-y)
Supplement: Supplementary file 1 — TABLE S1. Non-gender stratified multiple linear regression for associations of RBANS domain index and subtest scores with vitamin B12 and folate [file mmc1.docx]

**TABLE S1. Non-gender stratified multiple linear regression for associations of RBANS domain index and subtest scores with vitamin B12 and folate**

| RBANS Test scores | B12 | | Folate | |
| --- | --- | --- | --- | --- |
|  | β (95% CI) | p | β (95% CI) | p |
| Immediate Memory | 0.005 (-0.002, 0.011) | 0.189 | -0.034 (-0.085, 0.018) | 0.198 |
| List learning | 0.002 (0.000, 0.004) | 0.079 | -0.013 (-0.030, 0.004) | 0.124 |
| Story memory | 0.001 (-0.001, 0.003) | 0.374 | -0.003 (-0.016, 0.010) | 0.672 |
| Visuospatial | 0.000 (-0.007, 0.008) | 0.912 | 0.024 (-0.034, 0.083) | 0.414 |
| Figure copy | 0.000 (-0.002, 0.001) | 0.564 | 0.004 (-0.005, 0.014) | 0.360 |
| Line orientation | 0.000 (-0.001, 0.002) | 0.749 | 0.004 (-0.007, 0.014) | 0.503 |
| Language | -0.002 (-0.009, 0.005) | 0.663 | 0.010 (-0.043, 0.063) | 0.718 |
| Picture Naming | 0.000 (0.000, 0.001) | 0.503 | 0.002 (-0.002, 0.005) | 0.296 |
| Semantic fluency | -0.001 (-0.004, 0.001) | 0.285 | -0.002 (-0.021, 0.017) | 0.849 |
| Attention | -0.002 (-0.011, 0.006) | 0.563 | **-0.077 (-0.138, -0.015)** | **0.015** |
| Digit span | -0.001 (-0.002, 0.000) | 0.125 | **-0.018 (-0.028, -0.007)** | **0.001** |
| Coding | 0.001 (-0.004, 0.005) | 0.743 | -0.005 (-0.038, 0.027) | 0.743 |
| Delayed Memory | 0.003 (-0.003, 0.010) | 0.294 | 0.000 (-0.049, 0.049) | 0.992 |
| List recall | 0.001 (-0.001, 0.002) | 0.215 | -0.004 (-0.015, 0.007) | 0.453 |
| List recognition | 0.001 (0.000, 0.001) | 0.086 | 0.001 (-0.004, 0.006) | 0.638 |
| Story recall | 0.000 (-0.001, 0.001) | 0.778 | -0.002 (-0.010, 0.006) | 0.595 |
| Figure recall | -0.001 (-0.003, 0.001) | 0.438 | 0.006 (-0.008, 0.020) | 0.396 |
| RBANS Total Score | 0.001 (-0.006, 0.008) | 0.821 | -0.020 (-0.071, 0.031) | 0.437 |

Note: Adjusted for age, gender, educational attainment and BMI. Sensitivity analysis adjusting for SBP, DBP, cholesterol, creatinine and glucose did not change the results.

CI= confidence interval; P<0.05 in bold type
